# Analysis of the Correlation between Prognostic Nutritional Index and Diabetic Retinopathy in Patients with Diabetes

**DOI:** 10.2174/0118715303423094251129065818

**Published:** 2026-01-15

**Authors:** Qinyun Chen, Muye Li

**Affiliations:** 1 Department of Ophthalmology, Affiliated Hospital of North Sichuan Medical College, Nan Chong, 637000, Sichuan Province, China;; 2 Department of Vitreoretinopathy, Shanxi Eye Hospital, Taiyuan, 030002, China

**Keywords:** Prognostic nutritional index, diabetic retinopathy, non-linear relationship, nutritional status, inflammation, microvascular complication

## Abstract

**Introduction:**

This study aimed to investigate the correlation between Prognostic Nutritional Index (PNI) and diabetic retinopathy (DR) in patients with diabetes.

**Methods:**

In this cross-sectional NHANES study, 3,318 diabetic patients aged ≥40 years were analyzed. PNI was calculated as: serum albumin (g/L) + 5 × lymphocyte count (10^9^/L). Statistical analyses incorporated complex sampling survey design methods. Associations between DR and PNI were assessed using univariate and multivariate logistic regression models, with restricted cubic splines detecting potential non-linearity. Subgroup analyses examined population-specific effects.

**Results:**

Among the participants, 679 (18%) had DR. Patients with DR exhibited significantly lower PNI (50.46 ± 5.35) than those without DR (52.26 ± 6.07, *P* < 0.001). After adjusting for covariates—including age, sex, race, education, BMI, smoking status, hypertension, coronary heart disease, anemia, and blood glucose management—PNI remained inversely associated with DR risk (OR = 0.96, 95% CI: 0.94–0.98, *P* < 0.01). Restricted cubic spline analysis confirmed a non-linear relationship between PNI and DR (*P* = 0.01). Subgroup analyses showed significant inverse associations in patients aged 40–59 (OR = 0.93, 95% CI: 0.88–0.98, *P* = 0.01) and 60–79 (OR = 0.93, 95% CI: 0.89–0.96, *P* < 0.01), in overweight (OR = 0.94, 95% CI: 0.90–0.98, *P* < 0.01) and obese individuals (OR = 0.93, 95% CI: 0.89–0.96, *P* < 0.01), and in those using no medication (OR = 0.95, 95% CI: 0.90–0.99, *P* = 0.02) or oral hypoglycemic agents (OR = 0.96, 95% CI: 0.92–1.00, *P* = 0.04). The inverse association was not significant in patients with anemia or coronary heart disease (all quartiles *P* > 0.05).

**Discussion:**

This study demonstrates a significant non-linear inverse association between PNI and DR risk, particularly among adults aged 40–79 years and overweight/obese individuals, while the relationship was attenuated in those with anemia or coronary heart disease. As a composite nutritional-inflammatory marker, PNI may reflect overall health status in diabetic patients.

**Conclusion:**

PNI shows potential as a simple biomarker for DR risk stratification. Prospective validation is needed before clinical application.

## 1 INTRODUCTION

Diabetic retinopathy (DR)—a leading microvascular complication of diabetes—is a major global cause of vision loss and blindness in adults [1]. With the worldwide prevalence of diabetes continuing to rise, early detection and prevention of DR have become increasingly critical [2]. Although tight glycemic and blood pressure control are well-established strategies for DR prevention, a substantial proportion of patients still develop the condition despite adequate management [3, 4]. Consequently, identifying novel risk markers and potential therapeutic targets has emerged as a major focus of current research.

The role of nutritional status and chronic inflammation in the pathogenesis of DR has gained increasing attention in recent years [5]. The Prognostic Nutritional Index (PNI), an indicator reflecting an individual’s immune and nutritional status, was first proposed by Buzby *et al*. [6] and is calculated based on serum albumin levels and lymphocyte counts [7]. Originally developed to assess prognosis in patients undergoing gastrointestinal surgery or with cancer [8, 9], PNI has subsequently demonstrated predictive value in various chronic diseases [10-12].

These observations suggest a potential link between DR—a chronic microvascular disorder—and PNI, possibly reflecting shared pathways involving chronic inflammation or nutritional vulnerability. The components of PNI, namely serum albumin and lymphocyte count, likely play important roles in DR pathogenesis. Beyond reflecting nutritional status, serum albumin participates in physiological processes such as maintaining osmotic pressure and transporting hormones and fatty acids [13]. In diabetic patients, low serum albumin levels may contribute to increased microvascular permeability and retinal edema [14]. Lymphocytes, as key immune components, help regulate inflammatory responses and angiogenesis [15]. Thus, PNI, as a composite marker, may influence DR onset and progression through multiple pathways.

Notably, PNI may affect DR progression *via* retinal neovascularization—a hallmark of proliferative diabetic retinopathy (PDR) driven by an imbalance between pro-angiogenic and anti-angiogenic factors [16, 17]. Chronic inflammation and malnutrition may promote neovascularization by upregulating vascular endothelial growth factor (VEGF) [18]. Given that PNI reflects both inflammatory and nutritional status, it may be closely linked to this process. Furthermore, PNI could serve as an indicator of retinal microcirculatory function. Early DR is characterized by microvascular endothelial dysfunction in diabetic patients [19]. Serum albumin protects the endothelium by scavenging free radicals and preserving vascular barrier integrity [20], while lymphocytes modulate endothelial cell activation and apoptosis [21, 22].

Although some studies have explored the association between PNI and diabetic microvascular complications [23], the specific relationship between PNI and DR—particularly across diverse populations—remains poorly characterized. Key unanswered questions include whether PNI can serve as an independent risk marker for DR onset and progression, and its potential clinical utility in DR prevention and management.

Building on this evidence, the present study investigates the association between PNI and DR using the NHANES database, with a focus on potential variations across different population subgroups. We hypothesize that PNI may be associated with DR, potentially exhibiting non-linear characteristics. This research could provide novel insights for DR risk assessment and inform future preventive and therapeutic strategies.

## 2 MATERIALS AND METHODS

### 2.1 Study Design and Type of Study

This was a cross-sectional observational study using secondary analysis of public data from the National Health and Nutrition Examination Survey (NHANES).

#### 2.1.1 Participants and Data Sources

We obtained publicly available data from the NHANES (https://wwwn.cdc.gov/nchs/nhanes/Default.aspx). NHAN- ES is a nationally representative cross-sectional survey employing a stratified, multistage probability sampling design to collect health examination data from the non-institutionalized US population biennially. The survey combines self-reported and directly measured data through detailed household interviews (covering health status, behaviors, and dietary intake) and mobile examination center assessments, including blood sample collection.

The current analysis included data from 5 consecutive NHANES cycles (2009-2010 through 2017-2018), comprising an initial sample of 49,693 participants. After excluding 44,569 non-diabetic individuals, we identified 5,124 adults with diabetes. We further excluded 1806 participants who were aged <40 years or had missing data for independent variables (serum albumin, peripheral lymphocyte count), the dependent variable (diabetic retinopathy status), or important covariates. The final analytical sample included 3,318 eligible diabetic patients shown in Fig. (**1**), representing 21,040,300 weighted cases (predominantly Type 2 diabetes, based on age demographics). The final sample of 3,318 participants included 679 cases of diabetic retinopathy, providing an events-per-variable ratio of 61.7, which exceeds the recommended threshold of 10 events per variable for stable logistic regression analysis.

As NHANES received approval from the National Center for Health Statistics Research Ethics Review Board, no additional ethical approval/review was required.

#### 2.1.2 Definition of Diabetes and Diabetic Retinopathy

In our study, “diabetes” was defined as “diabetes informed by the doctor” or “diabetes not informed by the doctor”, and fasting blood glucose was greater than 7.0 mmol/L or glycated hemoglobin was greater than or equal to 6.5%. “Diabetic retinopathy” was defined by an affirmative response to the standardized questionnaire item: “Has a doctor ever told you that diabetes has affected your eyes or that you have retinopathy?” The answer is “Yes”.

#### 2.1.3 PNI Calculation

Haematological parameters were measured using the Beckman Coulter DxH 900 automated haematology analyser (Beckman-Coulter, Brea, California, USA) according to the NHANES complete blood count (CBC) profile protocol. The analyser performed red and white blood cell counts, haemoglobin measurement, haematocrit determination, and analysis of red blood cell indices. The Coulter VCS technology was used for white blood cell differential analysis. The Beckman Coulter analysis system performed sample processing using an automated dilution and mixing system, with hemoglobin quantification conducted by single-beam photometry. The PNI was calculated as: PNI = serum albumin (g/L) + (5 × lymphocyte count (10^9^/L)).

#### 2.1.4 Covariates

This study included the following covariates: age (categorised as 40-59, 60-79, and ≥80 years), sex (male/female), race/ethnicity (non-Hispanic white, non-Hispanic black, Mexican American, other Hispanic, other/multiracial), education level (<9^th^ grade, 9-11^th^ grade, high school graduate, some college/AA degree, college graduate or above), body mass index (categorised as underweight (<18.5 kg/m^2^), normal (18.5-25.0 kg/m^2^), overweight (25.0-30.0 kg/m^2^), and obese (>30.0 kg/m^2^), smoking status (never, former, current smoker). Laboratory measures included white blood cell count (10^9^/L), lymphocyte count (10^9^/L), neutrophil count (10^9^/L), serum albumin (g/L), and glycated haemoglobin (%). Hypertension, coronary heart disease and diabetes treatment regimens were self-reported *via* questionnaire. Anaemia was defined as haemoglobin <130 g/L for men and <110 g/L for women.

### 2.2 Statistical Analysis

All analyses were performed using R software version 4.4.1 (https://www.rstudio.com/products/rstudio/download). In accordance with NHANES analytical guidelines, we applied appropriate sampling weights to account for the complex survey design and ensure nationally representative estimates. Continuous variables with normal distributions are presented as mean ± standard deviation, while non-normally distributed variables are reported as median (interquartile range). Categorical variables are expressed as proportions or frequencies. Between-group comparisons were made using Rao-Scott second-order corrected χ^2^ tests for categorical variables and either one-way ANOVA or survey-weighted Wilcoxon rank-sum tests for continuous variables, as appropriate. We employed logistic regression models to examine risk factors, with statistical significance set at *P*<0.05. The relationship between DR and PNI was assessed using both univariable and multivariable logistic regression, with results expressed as odds ratios (ORs) and 95% confidence intervals (CIs). Restricted cubic splines were used to examine potential non-linear associations between PNI and DR.

## 3 RESULTS

### 3.1 Baseline Characteristics

Among the study participants, the distributions of sex, age, race/ethnicity, body mass index (BMI), smoking history, and hypertension showed no statistically significant differences between those with and without DR. Educational attainment differed significantly between groups (*P*<0.001). Those with DR were predominantly high school graduates or less educated (78%), while the non-DR group had higher education levels (33% college graduates and 22% with postgraduate qualifications). The mean prognostic nutritional index (PNI) was significantly lower in the DR group (50.46 ± 5.35) compared to non-DR participants (52.26 ± 6.07; *P*<0.001). The DR group showed a higher prevalence of both coronary heart disease (16% *vs* 8%) and anaemia (13% *vs* 5%). Participants with DR had significantly higher glycated haemoglobin (HbA1c) levels (*P*<0.001) and poorer glycaemic control. Those without DR demonstrated higher white blood cell, lymphocyte, and serum albumin levels (Table **1**).

### 3.2 Demographic and Clinical Characteristics of Participants Stratified by PNI Quartiles

The numbers of participants in each quartile were as follows: Q1 (n=924, 27%), Q2 (n=751, 23%), Q3 (n=842, 26%), and Q4 (n=801, 24%). In terms of demographic characteristics, including sex, age, race, and educational level, no significant differences were observed across quartiles in sex distribution or educational level (*P* > 0.9). However, age distribution differed significantly (*P* = 0.002), with 28% of participants in Q1 aged 40–59 years compared with 43% in Q4. Racial composition also varied significantly (*P* = 0.009), with non-Hispanic whites accounting for 64% in Q1 versus 57% in Q4. Regarding clinical characteristics, BMI, smoking status, glycaemic control method, diabetic retinopathy, coronary artery disease, and anaemia all showed statistically significant differences across PNI quartiles (Table **2**).

### 3.3 Univariable and Multivariable Logistic Regression Analyses of the Association between DR and PNI

**Fig. (2):**
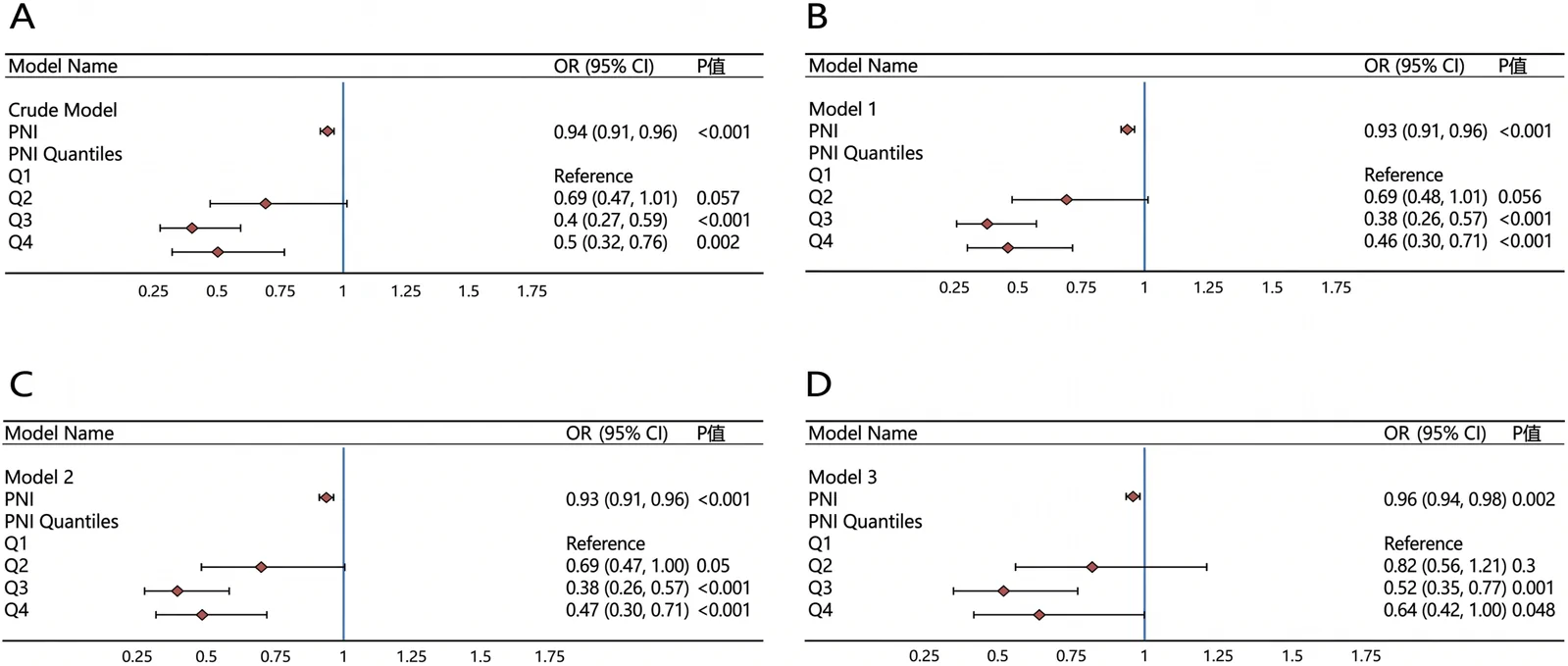
Univariable and multivariable logistic regression analyses of the association between DR and PNI. (**A**): Crude model; (**B**): Model 1, Adjusted for sex, age, race, and education. (**C**): Model 2, Adjusted for sex, age, race, education, BMI and smoking status; (**D**): Model 3, Adjusted for sex, age, race, education, BMI, smoking status, hypertension, CAD, anemia, and medication.

We performed univariable and multivariable logistic regression analyses to examine the association between diabetic retinopathy (DR) and the prognostic nutritional index (PNI) and its quartiles (Figs. **2A**-**D**). For the continuous PNI analysis, both univariable regression and adjusted models (Model 1 and Model 2) yielded similar odds ratio (OR) (0.94, 95% CI:0.91-0.96, *P*<0.001;0.93,95% CI:0.91-0.96, *P*<0.001;0.93,95% CI:0.91-0.96, *P*<0.001), all of which were statistically significant. Model 3 showed a slightly higher OR (0.96,95% CI:0.94-0.98, *P*=0.002), which remained statistically significant, though the association was weaker than in other models. When analyzing PNI quartiles, we used Q1 as the reference group. The Q2 group showed inconsistent statistical significance across models: nonsignificant in the univariable model (*P*=0.057), Model 2 (*P*=0.05), Model 1 (*P*=0.056), and Model 3 (*P*=0.3). In contrast, Q3 demonstrated statistically significant associations in all models, with OR ranging from 0.38 to 0.52, suggesting a robust inverse association. Similarly, Q4 showed consistent statistical significance across all models (OR: 0.46–0.64), supporting an inverse association. Overall, the results suggest that higher PNI may confer an inverse association for DR, though this association was slightly attenuated in Model 3. Notably, Q2 did not show a statistically significant association, whereas Q3 and Q4 exhibited significant inverse relationships in all models. These findings indicate that the association between DR and PNI may not be strictly linear.

### 3.4 Restricted Cubic Spline (RCS) Analysis of the Association between DR and PNI

**Fig. (3):**
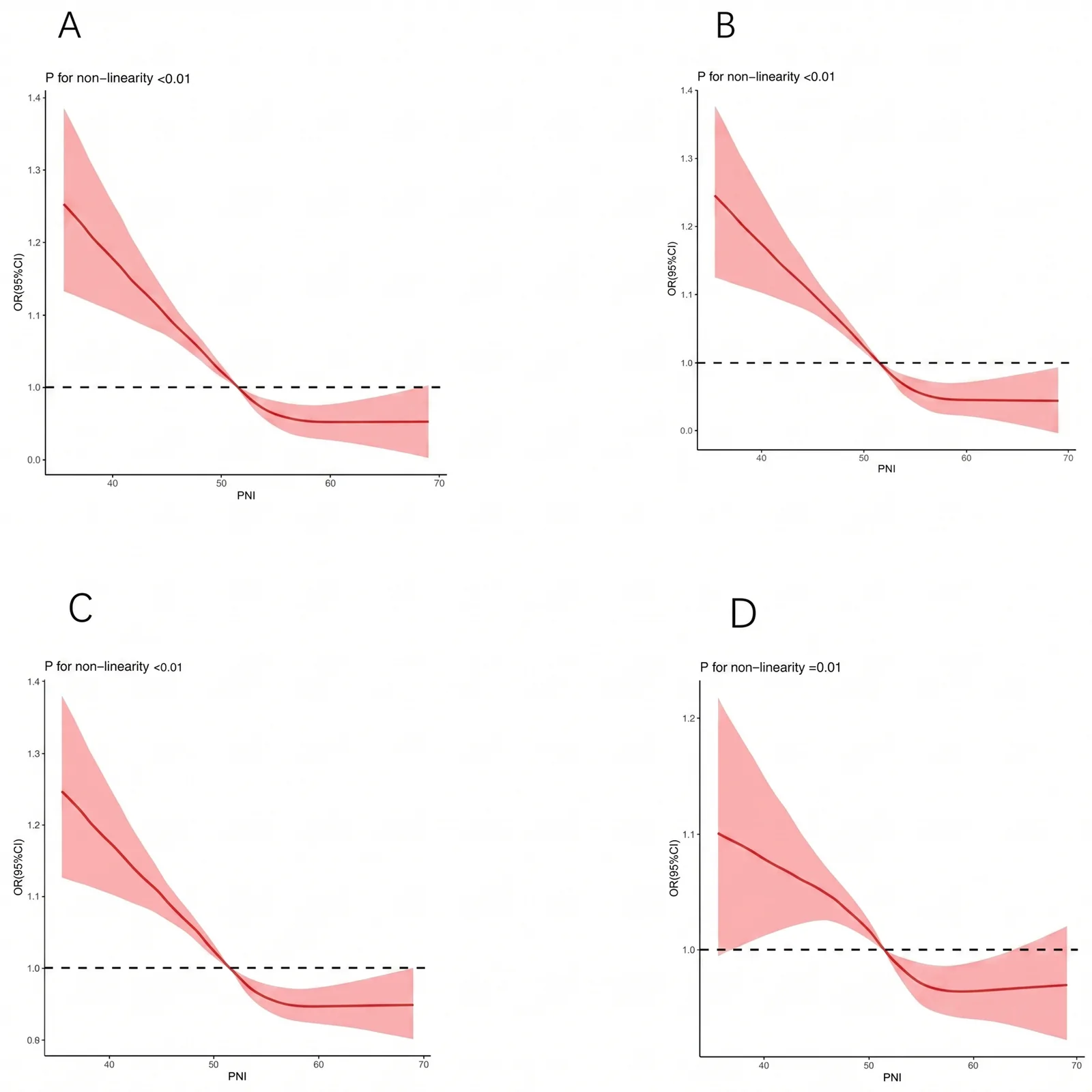
Restricted cubic spline (RCS) analysis of the association between DR and PNI. (**A**): Crude model; (**B**): Model 1, Adjusted for sex, age, race, and education. (**C**): Model 2, Adjusted for sex, age, race, education, BMI and smoking status; (**D**): Model 3, Adjusted for sex, age, race, education, BMI, smoking status, hypertension, CAD, anemia, and medication.

We performed restricted cubic spline analyses to flexibly model and visualize their association (Figs. **3A**-**D**). The results demonstrated a statistically significant non-linear relationship between DR and PNI (all P for non-linearity <0.01). This association remained significant after multivariable adjustment in Model 1 (*P*<0.01), Model 2 (*P*<0.01), and Model 3 (*P*=0.01). The curves demonstrate a threshold effect where DR risk decreases sharply when PNI increases from approximately 35-45, then plateaus at higher PNI levels (>50). This suggests a critical PNI threshold below which DR risk increases substantially.

### 3.5 Subgroup Analysis of the Effect of PNI on DR

Subgroup analyses revealed that both continuous PNI and PNI quartiles (Q3-Q4) showed significant inverse association in the 40-59 and 60-79 years age groups (all *P*<0.01), but not in those aged ≥80 years (Table **3A**). Continuous PNI demonstrated protection in non-Hispanic white (OR=0.92, 95% CI:0.89-0.98, *P*<0.01), non-Hispanic black (OR=0.96, 95% CI:0.93-0.98, *P*<0.01), and Mexican Americans (OR=0.94, 95% CI:0.91-1.00, *P*=0.05), but not in other/mixed ethnicities (OR=0.94, 95% CI:0.88-1.01, *P*=0.06). In quartile analyses, Q2 showed no significance across ethnic groups. Q3 exhibited protection in non-Hispanic whites (OR=0.26, 95% CI:0.13-0.50, *P*<0.01), and Mexican Americans (OR=0.41, 95% CI:0.19-0.90, *P*<0.03), but not in non-Hispanic Black, other Hispanics or mixed ethnicities. Q4 showed protection in non-Hispanic white (OR=0.46, 95% CI:0.23-0.91, *P*=0.03) and black (OR=0.45, 95% CI:0.26-0.77, *P*<0.01), but not in Mexican Americans or other/mixed ethnicities (Table **3B**). Continuous PNI showed protection across all education levels, being strongest in Bachelor's degree or higher (OR=0.90, 95% CI:0.83-0.97, *P*=0.01). For quartiles, Q3 protected those with high school education or below (OR 0.33-0.37), while Q4 protected those with some College or equivalent (OR=0.48, 95% CI:0.23-0.98, *P*=0.04) and Bachelor's degree or higher (OR=0.26, 95% CI:0.09-0.75, *P*=0.01) (Table **3C**). Significant effects were observed in overweight and obese groups (both *P*<0.01) but not in normal or underweight individuals. In obesity, Q2-Q4 all showed protection versus Q1 (all *P*<0.05), while in normal weight, only Q3 was inverse (*P*=0.03). Analysis of the underweight group was unfeasible due to the small sample size (Table **3D**). Medication-stratified analyses showed PNI's protection in no-medication and oral medication groups, but only Q3 showed protection in insulin users (Table **3E**). Smoking-stratified analyses revealed protection across all smoking status groups (all *P*<0.01), with Q3-Q4 inverse in never/current smokers and Q2-Q3 in former smokers (Table **3F**). Anemia- stratified analyses showed protection only in non-anemic individuals (*P*<0.01) (Table **3G**). Regardless of Coronary heart disease (CHD) status, continuous PNI showed protection (both *P*≤0.01), though quartile effects were only significant in the non-CHD group (Table **3H**). In summary, the protective association of PNI was most pronounced in adults aged 40–79 years and was consistent in non-Hispanic White and Black subgroups. More robust effects were observed in individuals with overweight or obesity, those not using medication or receiving oral medication agents, and likely enhanced among smokers. However, when analysed by quartiles, the protective effect was attenuated in patients with anaemia or coronary heart disease.

## 4 DISCUSSION

Using NHANES data, we investigated the association between the Prognostic Nutritional Index (PNI) and diabetic retinopathy (DR). Our findings demonstrate a significant nonlinear inverse relationship between PNI and DR, indicating that higher PNI levels are associated with a reduced risk of DR development. These results provide important insights for DR risk assessment and prevention strategies, highlighting the central role of nutritional-metabolic status and inflammatory responses in DR pathogenesis.

Our study shows that patients with lower PNI levels are significantly more likely to develop DR. After adjusting for potential confounders, PNI remained independently associated with DR risk in diabetic patients. This aligns with previous research by Yang *et al*., who reported an inverse relationship between PNI and both the prevalence and severity of DR in type 2 diabetes [24]. As a composite marker of nutritional and inflammatory status, reduced PNI likely reflects overall health deterioration, consequently increasing DR risk [7].

The two components of PNI—serum albumin and lymphocyte count—appear to play key roles in DR pathogenesis. Serum albumin levels were significantly lower in patients with DR compared to those without, consistent with evidence linking albumin deficiency to chronic diabetic microvascular complications [25]. Beyond serving as a nutritional marker, albumin is a crucial antioxidant; its reduction may impair cellular defenses against oxidative stress, exacerbating metabolic dysregulation and retinal dysfunction [13, 26-29]. Lymphocytes, essential for immune regulation, modulate inflammatory responses and vascular homeostasis; lymphopenia has been associated with DR development [30-33], further supporting the interplay between nutrition, inflammation, and microvascular complications in diabetes.

Type 2 diabetes is characterized by a chronic low-grade inflammatory state [34]. Insulin resistance triggers the production of pro-inflammatory cytokines and acute-phase proteins, such as C-reactive protein (CRP), plasminogen activator inhibitor-1 (PAI-1), and serum amyloid A, which are linked to microvascular complications [35-38]. PNI has also been associated with inflammatory conditions and long-term outcomes in diseases including cancer, COVID-19, and coronary artery disease [8, 39-44], positioning it as a novel inflammatory biomarker relevant to DR.

Multiple inflammatory and metabolic pathways—such as oxidative stress, advanced glycation end-product formation, the polyol and DAG-PKC pathways, and immune dysfunction—contribute to DR development [45-47]. Elevated inflammatory markers, including CRP, TNF-α, PAI-1, and soluble endothelial adhesion molecules, have been associated with DR progression [48-51]. A decline in PNI may thus reflect the inflammatory burden in DR, consistent with our findings that patients with DR exhibited lower PNI levels [52, 53]. Chronic inflammation can alter retinal cellular metabolism, promoting a glycolysis-dominant “Warburg effect”-like state in retinal pigment epithelial cells—a key feature of DR pathogenesis.

Our study revealed a non-linear relationship between PNI and DR, suggesting a potential threshold effect: DR risk may increase markedly when PNI falls below a critical level. This highlights the importance of maintaining adequate PNI for DR prevention, in line with similar non-linear associations reported for other diabetic complications, such as HbA1c and diabetic nephropathy [54].

Subgroup analyses showed that the inverse association between PNI and DR was strongest in patients aged 40–79 years, while attenuated in those over 80, likely due to complex health status in the elderly [55]. The association was consistent across non-Hispanic White and Black populations, indicating potential cross-racial applicability [56]. Overweight and obese individuals exhibited a more pronounced inverse association, reflecting the role of obesity as a chronic inflammatory state where PNI better captures metabolic and inflammatory risk [57-63].

When stratified by glycemic control strategy, the inverse association was more evident in patients using no medication or oral antidiabetic agents, whereas insulin-treated patients showed no significant effect, likely due to longer disease duration and multiple pathological factors influencing DR risk [3]. Smoking appeared to modulate the protective effect of higher PNI, with current smokers showing a more pronounced inverse association, possibly due to PNI counteracting smoking-induced endothelial injury [64].

The protective effect of PNI was attenuated in patients with anemia or coronary artery disease. Anemia may exacerbate retinal ischemia and influence nutritional status, while CHD-associated systemic inflammation may diminish PNI’s predictive value [65-71]. These findings underscore the multifactorial etiology of DR and the need to consider comorbidities in clinical management.

Strengths of this study include the use of large-scale, nationally representative survey data and the application of restricted cubic spline analysis to capture the non-linear relationship between PNI and DR. Limitations include reliance on self-reported DR diagnoses, the cross-sectional design preventing causal inference, potential residual confounding from unmeasured factors such as diet and physical activity, and the lack of evaluation of other diabetic microvascular and macrovascular complications [72-75].

Despite these limitations, our findings have clinical relevance: PNI is a simple, non-invasive, and readily obtainable marker with potential utility for DR risk stratification, particularly in middle-aged/older, overweight, or obese patients. Incorporating PNI into routine assessments may facilitate earlier identification of high-risk individuals for targeted preventive interventions.

## CONCLUSION

This large, population-based study demonstrates a significant nonlinear inverse association between PNI and diabetic retinopathy. The relationship varied across patient subgroups, being strongest in middle-aged adults (40–79 years) and in overweight or obese individuals, while attenuated in those with anemia or coronary heart disease. As a simple, readily obtainable biomarker derived from routine laboratory tests, PNI shows promise for improving DR risk stratification; however, prospective studies are needed to validate its clinical utility.

## Figures and Tables

**Fig. (1):**
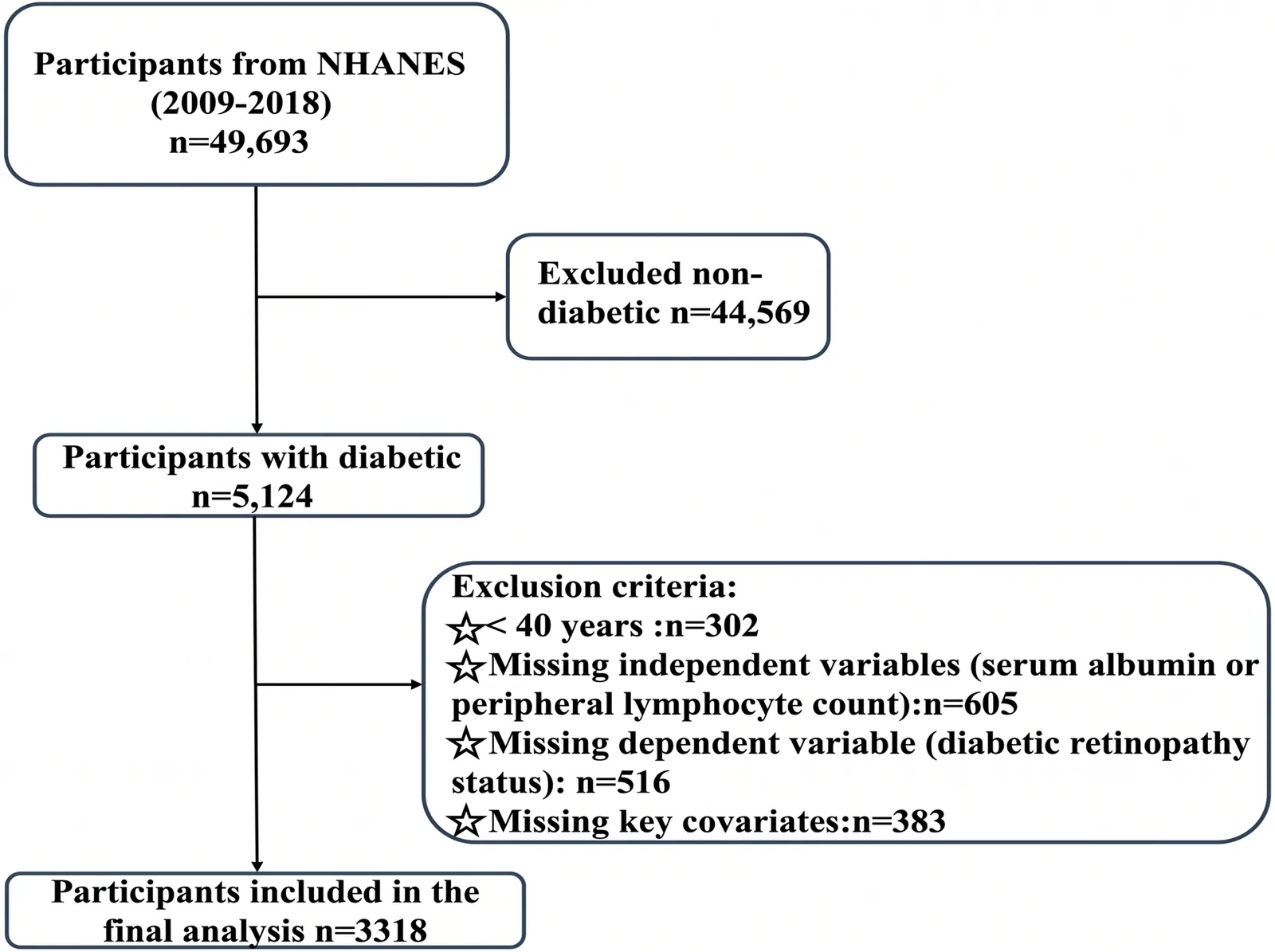
Flowchart of sample selection.

**Table 1:** Baseline characteristics.

**Characteristic**	**Overall**, N = 3318 (100%)^1^	**Diabetic Retinopathy**		***P* Value** ^2^
		**DR**, N = 679 (18%)^1^	**Non-DR**, N = 2639 (82%)^1^	
Sex	-	-	-	0.6
female	1,578 (47%)	313 (49%)	1,265 (47%)	-
male	1,740 (53%)	366 (51%)	1,374 (53%)	-
Age (years)	-	-	-	0.060
40-59 years	990 (36%)	205 (41%)	785 (34%)	-
60-79 years	1,708 (46%)	355 (39%)	1,353 (47%)	-
80+ years	620 (19%)	119 (19%)	501 (18%)	-
Race	-	-	-	0.3
Non-Hispanic White	1,107 (61%)	198 (58%)	909 (62%)	-
Non-Hispanic Black	831 (14%)	169 (15%)	662 (14%)	-
Mexican American	587 (9.6%)	116 (9.3%)	471 (9.7%)	-
Other/multiracial	430 (9.6%)	105 (11%)	325 (9.3%)	-
Other Hispanic	363 (5.7%)	91 (6.7%)	272 (5.4%)	-
Education	-	-	-	0.019
Less Than 9^th^ Grade	570 (9.6%)	126 (12%)	444 (9.2%)	-
9-11^th^ Grade	512 (12%)	115 (15%)	397 (12%)	-
High School Grad/GED	737 (24%)	154 (26%)	583 (24%)	-
Some College or AA degree	946 (33%)	191 (31%)	755 (33%)	-
College Graduate or above	553 (21%)	93 (15%)	460 (22%)	-
BMI (kg/m^2^)	-	-	-	0.9
Underweight (<18.5)	10 (0.2%)	1 (0.2%)	9 (0.2%)	-
Normal (18.5 to <25)	426 (11%)	98 (11%)	328 (11%)	-
Overweight (25 to <30)	966 (27%)	191 (28%)	775 (26%)	-
Obese (30 or greater)	1,916 (62%)	389 (61%)	1,527 (63%)	-
Smoking.Status	-	-	-	0.6
Never smoker	1,670 (49%)	340 (50%)	1,330 (49%)	-
Former smoker	1,145 (36%)	252 (37%)	893 (36%)	-
Current smoker	503 (15%)	87 (14%)	416 (16%)	-
Lymphocyte (10^9^/L)	2.15 (0.94)	2.00 (0.74)	2.19 (0.97)	<0.001
Neutrophil (10^9^/L)	4.79 (1.74)	4.68 (1.70)	4.82 (1.75)	0.094
WBC (10^9^/L)	7.82 (2.24)	7.53 (2.13)	7.89 (2.26)	0.007
HbA1c (%)	7.34 (1.69)	7.77 (1.76)	7.25 (1.66)	<0.001
Albumin (g/L)	41.2 (3.5)	40.4 (3.6)	41.3 (3.4)	<0.001
PNI	51.94 (5.98)	50.46 (5.35)	52.26 (6.07)	<0.001
Medication	-	-	-	<0.001
No	527 (16%)	66 (9.9%)	461 (18%)	-
Pills	1,861 (56%)	280 (36%)	1,581 (60%)	-
Insulin	408 (13%)	163 (30%)	245 (8.9%)	-
Both	522 (15%)	170 (24%)	352 (13%)	-
Hypertension	2,311 (69%)	499 (70%)	1,812 (69%)	0.8
CAD	381 (13%)	98 (16%)	283 (12%)	0.041
Anemia	441 (9.4%)	121 (13%)	320 (8.5%)	0.002

**Note:** ^1^median (IQR) for continuous; n (%) for categorical.^2^chi-squared test with Rao & Scott's second-order correction; Wilcoxon Rank-Sum test for complex survey samples.

**Table 2:** Demographic and clinical characteristics of participants stratified by PNI quartiles.

-		**PNI**				***P* Value** ^2^
**Characteristic**	**Overall**, N = 3318 (100%)^1^	**Q1**, N = 924 (27%)^1^	**Q2**, N = 751 (23%)^1^	**Q3**, N = 842 (26%)^1^	**Q4**, N = 801 (24%)^1^	
Sex	-	-	-	-	-	>0.9
female	1,578 (47%)	434 (47%)	336 (47%)	411 (48%)	397 (49%)	-
male	1,740 (53%)	490 (53%)	415 (53%)	431 (52%)	404 (51%)	-
Age (years)	-	-	-	-	-	0.002
40-59 years	990 (36%)	220 (28%)	221 (34%)	249 (38%)	300 (43%)	-
60-79 years	1,708 (46%)	483 (48%)	385 (47%)	462 (47%)	378 (40%)	-
80+ years	620 (19%)	221 (24%)	145 (18%)	131 (15%)	123 (16%)	-
Race	-	-	-	-	-	0.009
Non-Hispanic White	1,107 (61%)	346 (64%)	291 (66%)	248 (58%)	222 (57%)	-
Non-Hispanic Black	831 (14%)	250 (15%)	186 (13%)	209 (14%)	186 (14%)	-
Mexican American	587 (9.6%)	132 (7.5%)	124 (8.8%)	161 (10%)	170 (12%)	-
Other/multiracial	430 (9.6%)	99 (8.6%)	81 (7.4%)	127 (12%)	123 (10%)	-
Other Hispanic	363 (5.7%)	97 (5.4%)	69 (4.7%)	97 (5.7%)	100 (6.9%)	-
Education	-	-	-	-	-	0.3
Less Than 9^th^ Grade	570 (9.6%)	137 (8.1%)	126 (9.0%)	144 (10%)	163 (11%)	-
9-11^th^ Grade	512 (12%)	166 (16%)	109 (11%)	115 (9.9%)	122 (13%)	-
High School Grad/GED	737 (24%)	217 (25%)	161 (22%)	191 (26%)	168 (24%)	-
Some College or AA degree	946 (33%)	252 (30%)	240 (37%)	240 (33%)	214 (31%)	-
College Graduate or above	553 (21%)	152 (21%)	115 (21%)	152 (22%)	134 (21%)	-
BMI (kg/m^2^)	-	-	-	-	-	0.042
Underweight (<18.5)	10 (0.2%)	6 (0.6%)	0 (0%)	3 (0.2%)	1 (<0.1%)	-
Normal (18.5 to <25)	426 (11%)	125 (13%)	92 (8.6%)	108 (8.9%)	101 (12%)	-
Overweight (25 to <30)	966 (27%)	227 (23%)	225 (29%)	270 (26%)	244 (29%)	-
Obese (30 or greater)	1,916 (62%)	566 (63%)	434 (62%)	461 (65%)	455 (59%)	-
Smoking.Status	-	-	-	-	-	0.002
Never smoker	1,670 (49%)	456 (50%)	389 (52%)	413 (46%)	412 (48%)	-
Former smoker	1,145 (36%)	360 (38%)	266 (36%)	289 (39%)	230 (30%)	-
Current smoker	503 (15%)	108 (11%)	96 (13%)	140 (15%)	159 (22%)	-
Lymphocyte (10^9^/L)	2.15 (0.94)	1.52 (0.50)	1.88 (0.44)	2.24 (0.52)	3.04 (1.28)	<0.001
Neutrophil (10^9^/L)	4.79 (1.74)	4.77 (1.92)	4.67 (1.72)	4.87 (1.65)	4.87 (1.65)	0.071
WBC (10^9^/L)	7.82 (2.24)	7.11 (2.25)	7.38 (1.97)	8.00 (2.01)	8.87 (2.31)	<0.001
HbA1c (%)	7.34 (1.69)	7.43 (1.77)	7.32 (1.65)	7.18 (1.58)	7.45 (1.74)	0.2
Albumin (g/L)	41.2 (3.5)	38.1 (3.2)	40.9 (2.2)	42.2 (2.7)	43.8 (3.0)	<0.001
DR	-	-	-	-	-	<0.001
DR	679 (18%)	244 (NA%)	164 (NA%)	140 (NA%)	131 (NA%)	-
Non-DR	2,639 (82%)	680 (NA%)	587 (NA%)	702 (NA%)	670 (NA%)	-
Medication	-	-	-	-	-	<0.001
No	527 (16%)	139 (14%)	114 (14%)	141 (17%)	133 (20%)	-
Pills	1,861 (56%)	436 (47%)	419 (58%)	517 (62%)	489 (58%)	-
Insulin	408 (13%)	189 (21%)	101 (15%)	67 (7.6%)	51 (7.3%)	-
Both	522 (15%)	160 (17%)	117 (14%)	117 (14%)	128 (15%)	-
Hypertension	2,311 (69%)	676 (71%)	527 (70%)	571 (67%)	537 (68%)	0.7
CAD	381 (13%)	147 (19%)	82 (12%)	90 (10%)	62 (8.6%)	<0.001
Anemia	441 (9.4%)	239 (20%)	89 (8.2%)	68 (5.5%)	45 (3.3%)	<0.001

**Note:** ^1^median (IQR) for continuous; n (%) for categorical.^2^chi-squared test with Rao & Scott's second-order correction; Wilcoxon rank-sum test for complex survey samples.

**Table 3A:** Regression results by age group.

Age (Years)	-	**OR (95% CI)**	***P* Value**
**40-59**	PNI	0.93(0.88,0.98)	0.01
	Q1	Reference values	-
	Q2	0.40(0.19, 0.82)	0.01
	Q3	0.27(0.13, 0.53)	<0.01
	Q4	0.38 (0.18, 0.81)	0.01
**60-79**	PNI	0.93(0.89,0.96)	<0.01
	Q1	Reference values	-
	Q2	0.85(0.51,1.42)	0.53
	Q3	0.46(0.29,0.74)	<0.01
	Q4	0.48(0.27,0.88)	0.02
**80+**	PNI	0.95(0.90,1.00)	0.07
	Q1	Reference values	-
	Q2	1.02(0.47,2.22)	0.95
	Q3	0.51(0.23,1.33)	0.10
	Q4	0.56(0.26,1.20)	0.13

**Table 3B:** Regression results by race.

Race	-	**OR (95% CI)**	***P* Value**
**Non-Hispanic White**	PNI	0.92(0.89,0.98)	<0.01
	Q1	Reference values	-
	Q2	0.61(0.35, 1.08)	0.09
	Q3	0.26(0.13, 0.50)	<0.01
	Q4	0.46(0.23,0.91)	0.03
**Non-Hispanic Black**	PNI	0.96(0.93,0.98)	<0.01
	Q1	Reference values	-
	Q2	0.93(0.56,1.55)	0.78
	Q3	0.76(0.48, 1.19)	0.22
	Q4	0.45(0.26,0.77)	<0.01
**Mexican Americans**	PNI	0.96(0.91,1.00)	0.05
	Q1	Reference values	-
	Q2	0.55(0.23,1.32)	0.17
	Q3	0.41(0.19,0.90)	0.03
	Q4	0.63(0.31,1.28)	0.19
**Other Hispanics**	PNI	0.94(0.89,0.99)	0.02
	Q1	Reference values	-
	Q2	1.49(0.74,3.00)	0.25
	Q3	0.55(0.22,1.35)	0.18
	Q4	0.52(0.24,1.12)	0.09
**Other/mixed ethnicities**	PNI	0.94(0.88,1.01)	0.06
	Q1	Reference values	-
	Q2	0.78(0.35,1.76)	0.54
	Q3	0.73(0.30,1.78)	0.48
	Q4	0.58(0.27,1.26)	0.17

**Table 3C:** Regression results by education level.

Education Level	-	**OR (95% CI)**	***P* Value**
**<9^th^ grade**	PNI	0.95(0.91,0.99)	0.02
	Q1	Reference values	-
	Q2	0.88(0.44, 1.75)	0.71
	Q3	0.88(0.47, 1.65)	0.68
	Q4	0.56(0.27,1.17)	0.12
**9-11^th^ grade**	PNI	0.94(0.90,0.99)	0.01
	Q1	Reference values	-
	Q2	0.75(0.35, 1.63)	0.46
	Q3	0.37(0.17, 0.82)	0.02
	Q4	0.47(0.21,1.02)	0.05
**High school grade**	PNI	0.94(0.89,0.98)	0.01
	Q1	Reference values	-
	Q2	0.73(0.41,1.33)	0.30
	Q3	0.33(0.160.67)	<0.01
	Q4	0.70(0.32,1.56)	0.38
**College or equivalent**	PNI	0.95(0.91,0.99)	0.01
	Q1	Reference values	-
	Q2	0.70(0.37,1.30)	0.25
	Q3	0.35(0.19,0.62)	<0.01
	Q4	0.48(0.23,0.98)	0.04
**Bachelor's degree or higher**	PNI	0.90(0.83,0.97)	0.01
	Q1	Reference values	-
	Q2	0.55(0.19,1.57)	0.26
	Q3	0.41(0.14,1.23)	<0.11
	Q4	0.26(0.09,0.75)	0.01

**Table 3D:** Regression results by BMI.

BMI	-	**OR (95% CI)**	***P* Value**
**Normal (18.5 to <25)**	PNI	0.97(0.91,1.03)	0.36
	Q1	Reference values	-
	Q2	0.80(0.36, 1.78)	0.57
	Q3	0.40(0.18, 0.89)	0.03
	Q4	0.82 (0.34,2.02)	0.67
**Underweight (<18.5)**	PNI	1.13(NA,NA)	0.32
	Q1	Reference values	-
	Q2	NA	NA
	Q3	NA	NA
	Q4	NA	NA
**Overweight (25 to <30)**	PNI	0.94(0.90,0.98)	<0.01
	Q1	Reference values	-
	Q2	0.85(0.41,1.76)	0.65
	Q3	0.46(0.24,0.89)	0.02
	Q4	0.50(0.24,1.08)	0.08
**Obese (30 or greater)**	PNI	0.93(0.89,0.96)	<0.01
	Q1	Reference values	-
	Q2	0.60(0.39,0.93)	0.02
	Q3	0.36(0.21,0.61)	<0.01
	Q4	0.43(0.24,0.78)	0.01

**Abbreviation:** NA: Not Available.

**Table 3E:** Regression results by medication.

Medication	-	**OR (95% CI)**	***P* Value**
**No**	PNI	0.95(0.90,0.99)	0.02
	Q1	Reference values	-
	Q2	1.17(0.45, 3.08)	0.75
	Q3	0.89(0.37,2.16)	0.79
	Q4	0.37((0.17,0.81)	0.01
**Pills**	PNI	0.96(0.92,1.00)	0.04
	Q1	Reference values	-
	Q2	0.95(0.57, 1.60)	0.86
	Q3	0.55(0.33, 0.90)	0.02
	Q4	0.86((0.47,1.56)	0.61
**Insulin**	PNI	0.95(0.90,1.00)	0.05
	Q1	Reference values	-
	Q2	0.66(0.28,1.56)	0.34
	Q3	0.28(0.13,0.63)	<0.01
	Q4	0.54(0.22,1.30)	0.17
**Both**	PNI	0.97(0.92,1.03)	0.31
	Q1	Reference values	-
	Q2	0.55(0.23,1.34)	0.18
	Q3	0.54(0.22,2.16)	0.16
	Q4	0.61(0.26,1.44)	0.26

**Table 3F:** Regression results by smoking status.

Smoking Status	-	**OR (95% CI)**	***P* Value**
**No**	PNI	0.94(0.91,0.98)	<0.01
	Q1	Reference values	-
	Q2	0.83(0.55, 1.24)	0.36
	Q3	0.40(0.24, 0.65)	<0.01
	Q4	0.63(0.35, 1.12)	0.11
**Yes, Current Smoker**	PNI	0.91(0.86,0.97)	<0.01
	Q1	Reference values	-
	Q2	0.74(0.24,2.31)	0.60
	Q3	0.35(0.16,0.75)	0.01
	Q4	0.26(0.11,0.61)	<0.01
**Yes, Former Smoker**	PNI	0.94(0.90,0.98)	<0.01
	Q1	Reference values	-
	Q2	0.51(0.29,0.92)	0.03
	Q3	0.41(0.22,0.78)	0.01
	Q4	0.49(0.24,1.02)	0.06

**Table 3G:** Regression results by anemia.

Anemia	-	**OR (95% CI)**	***P* Value**
**No**	PNI	0.94(0.91,0.97)	<0.01
	Q1	Reference values	-
	Q2	0.72(0.47, 1.10)	0.12
	Q3	0.41(0.27, 0.64)	<0.01
	Q4	0.52(0.32, 0.84)	0.01
**Yes**	PNI	0.94(0.89,0.98)	0.01
	Q1	Reference values	-
	Q2	0.70(0.32,1.53)	0.37
	Q3	0.45(0.18,1.14)	0.09
	Q4	0.44(0.18,1.12)	0.08

**Table 3H:** Regression results by CAD.

CAD	-	**OR (95% CI)**	***P* Value**
**No**	PNI	0.94(0.91,0.97)	<0.01
	Q1	Reference values	-
	Q2	0.72(0.47, 1.10)	0.12
	Q3	0.41(0.27, 0.64)	<0.01
	Q4	0.52(0.32, 0.84)	0.01
**Yes**	PNI	0.94(0.89,0.98)	0.01
	Q1	Reference values	-
	Q2	0.70(0.32,1.53)	0.37
	Q3	0.45(0.18,1.14)	0.09
	Q4	0.44(0.18,1.12)	0.08

## Data Availability

The data that support the findings of this study are openly available in NHANES at https://wwwn.cdc.gov/nchs/nhanes/Default.aspx.

## LIST OF ABBREVIATIONS

DR: Diabetic Retinopathy
PNI: Prognostic Nutritional Index
PDR: Proliferative Diabetic Retinopathy
VEGF: Vascular Endothelial Growth Factor
NHANES: National Health and Nutrition Examination Survey
CBC: Complete Blood Count

## STANDARDS OF REPORTING

STROBE guidelines were followed.
